# Supplementary material for: Learning curve and surgical outcome of robotic assisted colorectal surgery with ERAS program
Source: Sci Rep. 2022 Nov 29;12:20566. doi: 10.1038/s41598-022-24665-w (PMC9709162; doi:10.1038/s41598-022-24665-w)
Supplement: Supplementary file 1 — Supplementary Information 1. [file 41598_2022_24665_MOESM1_ESM.zip › LSC Raw data-Ñ[▒K/058/HS_ñw╝╨░O▒Kñσ.pdf]

正式摘要(電子簽章：0000001831 23974)		
W83 / W83 ☐ 英文標題(注意:無英文姓名-請補病人基本檔!)	☐【讀取】憑證	

臺中榮民總醫院
出院病歷摘要

轉入號： 轉出號：

[redacted]

(8)流水編號	
(9)入院日期	108/12/16 (2019) 直腸外科 W83 - 115
(10)轉科日期	
(11)出院日期	108/12/31 (2019) 住院天數計 15 日

診斷

- 入院：1. Recurrent acute pancreatitis with fluid accumulation over peri-pancreatic head region, s/p Flomoxef 10/20-10/30
  2. Avascular necrosis of right femoral head associated with alcholism. CT: Subchondral fracture and collapse at R't femoral head
  3. Rectal tumor, about 3.5cm found by abdominal CT, R/O rectal cancer, s/p sigmoidoscopy with biopsy. Pathology reported tubulovillous adenoma
  4. Gout
- 出院：1. Acute pancreatits in 2019/2, hospitalized at 署立豐原, discharged around 2019/03,04
  2. Recurrent acute pancreatitis with fluid accumulation over peri-pancreatic head region, s/p Flomoxef 10/20-10/30
  3. Traffic injury s/p STSG 12 year ago.
  4. Avascular necrosis of right femoral head associated with alcholism. CT: Subchondral fracture and collapse at R't femoral head
  5. Rectal Tubulovillous adenoma with focal high grade dysplasia., s/p laparoscopic LAR + loop ileostomy on 20191217
  6. Gout s/p chlochicine

主訴

- diarrhea for 6 months and constipation and abdominal pain for recently

病史

- This 40 y/o patient had the underlying of
  1. Acute pancreatits in 2019/2, hospitalized at 署立豐原, discharged around 2019/03,04
  2. Alcoholism, quitted in 2019/2
  3. Traffic injury s/p STSG 12 year ago.
  4. Recurrent acute pancreatitis with fluid accumulation over peri-pancreatic head region, s/p Flomoxef 10/20-10/30

  He complained about abdominal pain started at 2019/10/17, accompanied with nausea and vomiting after drinking water. The vomitus was greenish. Therefore, he stopped eating and came to 署立豐原. He was referred to our ER on 10/18 due to persistance of discomfort.
  At ER, Lab data showed leukocytosis with left shift (81%) and elevation of CRP. Abdominal CT revealed dilatation of pancreatic head with fluid accumulation and peripancreatic fat stranding, necrotizing pancreatitis may be compatible and a 3.5cm mass lesion at rectum, suggest coloscopy to R/O tumor. Therefore, he was transferred to GI ward on 10/20.
  In hospitalization, Flomoxef was prescribed for pancreatitis. Sigmoidoscopy with biopsy was done on 10/21 which revealed a tubulovillous adenoma. Right hip joint pain was also mentioned, bil hip xray on 10/28 showed R/O right femoral head AVN. After discharged under stable condition on 10/30, he was f/u at our CRS and ORTH OPD.
  He admitted on 11/25 for laparoscopic low anterior resection, however pre-OP survey found leukocytosis and elevated CRP.
  After discussion with patient, laparoscopic LAR was re-arranged on 12/17.
  Therefore, he was admission for scheduled laparoscopic LAR on 12/17 this time.

體檢發現

- rectum tumor 8 cm FAV.
  - General appearance :
    - easy looking , conscious clear , without cardiopulmonary distress

-
  - Vital signs自動帶入date 體溫(BT) 脈搏(P) 呼吸(R) 血壓(BP) 身高(BH) 體重(BW) BMI
    20191125 36.3℃ 92次/分 18次/分 126/86mmHg 184.5cm 63kg 18.5
  - Vital signs: B.P. 126/86 mmHg , P.R. 92 /min , R.R. 18 /min , B.T. 36.3 ℃
- HEENT :
  - Eyes: no anemic conjunctiva, no icteric sclera, normal EOM
  - ENT: no otorrhea, no rhinorrhea, no erythematous mucosa, no injected tonsil, no deformity
- Neck: supple, freely movable, trachea midline,no deformity, no engorged jugular vein , no palpable thyroid or lymph node enlargement
- Chest:
  - Inspection: Grossly normal, symmetric and fully expansion, no accessory muscle used, no operation scar, no spider angiomata, no gynecomastia
  - Palpation: no palpable mass or nodule, no subcutaneous emphysema, no tenderness
  - Ausculation: clear breathing sound
- Heart:
  - Palpation: no thrill, no heave
  - Percussion: normal heart size
  - Ausculation: regular heart beat, normal S1, S2, no gallop, no murmur
- Extremities: No deformity, full ranged of motion without limitation, no clubbing finger or nail cyanosis, no pitting edema, no ulcer or pigmentation, no obvious skin lesion, no varicose vein, no bone, joint or muscle tenderness

CRS CONDITION

- Karnofsky performance status: (100) Normal, no complaints, no evidence of disease
- PE of Abdomen:
- CEA: 4.3 ng/ml
- Imaging Studies:
- Colonoscope report: Colonoscopy was and up to Sigmoid colon . 1 polypoid lesion over 8cm AAV,pathology revealed Tubulovillous adenoma
- Figures:
  1. colon & rectum

手術日期及方法

- 手術日期及方法20191217 1. Laparoscopic assisted low anterior resection with end-to-end anastomosis, auto-EndoGIA 60/4.1mm*2, CDH 33mm, re-inforcement of silk (-), air leak test : no leakage.
  2. High ligation IMA above left colic artery.
  3. Cut margin: proximal 11 cm, distal 2 cm.
  4. Take down of splenic flexure: (-)
  5. A Fr.12 Penrose drain placed in the pelvis.
- 手術結果及發現 :
  - 1. One 3x3cm Borrmann type I1 tumor at middle rectum, which invaded to the mucosa layer but didn't penetrate through the submucosa. No enlarged lymph nodes at mesorectum were noted, cT1N0M0. 2. There was no palpable liver metastasis, no visible peritoneal seeding.

住院治療經過

- After admission, pre-op survey was done. Lab data showed chronic pancreatitis still presence. Low anterior resection with colo-anal anastomosis was done smoothly on 12/17. His Hb dropped to 6.7 on 12/18, blood transfusion was done without allergy reaction. However he suffered from gout attack on 12/19 with fever to 39.1, severe pain and swelling at r't knee. After ketorolac and methasone givan, his gout sign was relived. 12/23, He started to try water, and chlochicine was prescribed for 3 days. Penrose drainage was removed on 12/27, and stitches were removed on 12/30. There were plenty amount of feces from stoma after surgery, Loperamide was increased to 2# Q8H and Smecta 1# BID. He recovered well recently without any discomfort. He was discharged today under stable condition and f/u at OPD on 2020/1/7.

合併症

一般檢查

- 累積報告 :

DATE	TIME	ALB	TP	BIL.T	BIL.D	ALKP	AST	ALT	LDH	CHOL	TG	HDL-C	LDL	UA	NH3	AMY
1081118	1214							22								
1081125	1538	4.1		0.33	0.12	178	11	9	132							
1081126	1145															74

1081216	1901	4.2		0.3	0.1		11	13								141
1081218	0943	3.4		0.84	0.25		9	8								58
1081218	2326	2.8		0.5	0.2		10	7								
1081219	1612	3.1												8.4		59
1081221	0935	3.5														
1081223	1243	3.9		0.74	0.24		6	4								
1081224	0845													8.4		
1081226	0923	3.5		0.49				3								
1081230	0935	3.8		0.34				4						8.6		

DATE	TIME	WBC	RBC	HGB	HCT	MCV	PLT	BLST	NEUT	BAND	SEG	LYM	MON	EOS	BAS
1081118	1213	11800		14.0			397		72.2			20.3	6.7	0.5	0.3
1081125	1526	13620	4.03	12.1	38.0	94.3									
1081126	1134	10090	3.79	11.6	35.7	94.2	277		69.1			22.4	6.1	1.9	0.5
1081216	1911	14890	4.19	13.4	40.8	97.4	281		66.1			27.3	5.0	1.3	0.3
1081218	0943	20570		9.4			247		86.0			8.1	5.7	0.1	0.1
1081218	2326	16560	2.15	6.9			193		82.7			11.0	5.6	0.5	0.2
1081219	0301				20.7										
1081219	0906			8.4	24.8		142								
1081219	1613	14060		8.5			142		86.7			6.7	5.4	1.1	0.1
1081220	0856	13550		8.1			151		87.8			6.3	5.8	0.0	0.1
1081221	0917	10850		8.6			187		82.9			11.8	5.0	0.1	0.2
1081223	1319	11790	3.11	10.0	30.0	96.5	286		84.1			10.3	5.4	0.1	0.1
1081226	1028	17840	3.08	9.5			388			1.0	74.0	15.0	6.0	3.0	0.0
1081230	0910	13580	3.34	10.7	32.4	97.0	556		64.4			22.2	5.8	7.0	0.6

DATE	TIME	NA	K	CL	CA	BUN	CREAT	Mg	P	CRP	ESR	PCT	LIPASE	LACTATE
1081118	1214	140	4.1				0.87			0.468			94	
1081125	1538	145	3.8	108	9.3	10	0.81							
1081126	1122													7.0
1081126	1143											0.02	39	
1081126	1145									3.191				
1081216	1901	141	4.1	105	9.1	13	0.96						129	
1081218	0900													21.7
1081218	0943	137	4.6	104	8.5	17	1.16			7.721				
1081218	0952												32	
1081218	2326	138	3.8		7.9	20	0.92							8.7
1081219	1608													6.2
1081219	1631												34	
1081220	0854	139	4.2	108	9.2	12	0.83							
1081221	0935	137	4.3	105	9.4	15	0.74							
1081223	1243	138	4.5	110	9.5	21	0.78							
1081226	0923	141	4.6	114	9.1	20	0.80							
1081230	0935	143	3.9	112	9.4	21	0.89							

DATE	TIME	GLUCOSE	PAB	TRF	ZN	CU	KETONE	CK	CKMB	PT-P	PT-C	APTT-P	APTT-C	TRO-T
1081118	1215													8.77
1081125	1526									10.4	10.1	29.0		
1081125	1546	65												
1081216	1901									9.7	10.1	26.0		
1081218	0654								4					6.89
1081219	0933									11.0	10.1	28.8		

DATE	TIME	PH	BEB	SCVO2	PO2	HCO3	HGB	PCO2	TCO2
1081118	1212	7.372	0.8	68.7	37.0	26.6		46.8	28.0
1081218	0652	7.374	-3.4	93.6	72.0	21.3		37.3	22.4
1081219	1609	7.429	-0.9	90.1	57.1	22.9		35.4	24.0

- 放射線報告 :**檢驗項目 :ABDOMEN (KUB),SUPINE 報告日期 :1081223**

主　訴：
Result:
Finding:
> Much bowel gas and fecal material is noted.
> Degenerative joint disease over thoracic and lumbar spine
> Bilateral psoas and renal shadows are obscured by bowel gas.
> Avascular necrosis of the right femoral head.
> Surgical clips over central abdomen and abdomen
> Post drainage tube insertion over left pelvis
> Surgical stitches over pelvis
Impression:
As above description.

特殊檢查

病理報告

- 活體組織切片病理報告　手術日期：**108/12/18**　病理號：**S0845294**

診斷醫師:陳志榮 報告日期:108/12/20
Pathologic diagnosis:
1. Rectum, laparoscopic low anterior resection --- Tubulovillous adenoma with focal high grade dysplasia. The bilateral cut ends and perirectal lymph nodes (0/25) are free of lesion.
2. Rectum, cut end, distal, resection --- Chronic inflammation.
Prognostic and predictive factor:
1. Degree of dysplasia: High grade.
2. Margin of stalk: Free.
Gross description:
The specimen consists of 1) a segment of colon, 11.5 cm in length and 6 cm in proximal cut end circumference, 8.5 cm in distal circumference, and perirectal soft tissue. A gray tan polypoid tumor, 3.8x2.7 cm, is noted, 2.5 cm to the nearest cut end. On cross section, no definite stromal invasion is idnetified. The both cut ends and radial margin are free of tumor grossly. 2) distal cut end, a piece of tan mucosa-covered soft tissue, 2.1x1.7x0.7 cm.
Representative part for section: A) proximal cut end B) distal cut end C) colon D-F) tumor G) specimen 2 H-K) lymph node.
#T-59300_2 #M-82630_2 0 1105 000000
#T-C4520_2 #M-09183_2 0 1105 000000

活體組織切片病理報告　手術日期：**108/11/14**　病理號：**S0840704**

診斷醫師:孫志豪 報告日期:108/11/18
Pathologic diagnosis:
Rectum, 8 cm from anal verge, labeled "tumor", endoscopic biopsy --- Tubulovillous adenoma (see description).
Prognostic and predictive factor:
1. Degree of dysplasia: Low grade (moderate dysplasia).
2. Margin of stalk: Not free.
Gross description:
The specimen consists of multiple pieces of tan soft tissue, up to 0.2x0.2x0.2 cm. All for section.
Microscopic description:
Section shows colon mucosa with tubulovillous glands proliferation with moderate dysplasia or low grade dysplasia. Limited specimen may not be representative for the whole lesion. Rebiopsy is recommended if malignancy is suspected clinically.
Comment:
Further clinicopathological correlation and study are suggested.
#T-59300_2 #M-82630_2 0 1340 000000

活體組織切片病理報告　手術日期：**108/10/21**　病理號：**S0837254**

診斷醫師:詹以吉 報告日期:108/10/22
Pathologic diagnosis:
Rectum, endoscopic biopsy --- Tubulovillous adenoma.
Prognostic and predictive factor:
1. Degree of dysplasia: Low grade.
2. Margin of stalk: Cannot be well evaluated.
Gross description:
The specimen consists of 4 pieces of tan soft tissue, up to 0.3x0.1x0.1 cm. All for section.
Microscopic description:
Section shows colon mucosa with tubulovillous glands proliferation with dysplasia.
#T-59300_2 #M-82630_2 0 1340 000000

其它

出院時情況

- 3.改門診治療

出院指示

-

藥名	劑量	劑量單位	用法	頻次	數量	數量單位	現況	天數	單位
Loperamide cap 2mg	1	CAP	PO	QIDPRN	28	CAP	TH	7	天
$Biofermin tab	2	TAB	PO	TID	42	TAB	TH	7	天
Smecta powder 3g	1	PKG	PO	BID	14	PKG	TH	7	天
Voren-K tab 25mg	1	TAB	PO	TIDPRN	21	TAB	TH	7	天

出院計劃

- 門診日期
  - date on 2020/01/07

主治醫師 2222H 林俊余 / 住院醫師 9981E 蔡采錚

正式摘要**(電子簽章：000000183123974)**

W83 / W83

doctorRank:B
